# Reduced levels of CCL2 and CXCL10 in systemic lupus erythematosus patients under treatment with prednisone, mycophenolate mofetil, or hydroxychloroquine, except in a high STAT1 subset

**DOI:** 10.1186/ar4451

**Published:** 2014-01-24

**Authors:** Paul R Dominguez-Gutierrez, Angela Ceribelli, Minoru Satoh, Eric S Sobel, Westley H Reeves, Edward KL Chan

**Affiliations:** 1 Department of Oral Biology, University of Florida, 1395 Center Drive, Gainesville, FL 32610-0424, USA; 2 Division of Rheumatology and Clinical Immunology, Department of Medicine, University of Occupational and Environmental Health, Japan, 1-1 Isei-ga-oka, Yahata-nishi-ku, Kitakyushu, Fukuoka 807-8555, Japan; 3 School of Health Sciences, University of Occupational and Environmental Health, Japan, 1-1 Isei-ga-oka, Yahata-nishi-ku, Kitakyushu, Fukuoka 807-8555, Japan; 4 Current address: Rheumatology and Clinical Immunology, Humanitas Clinical and Research Center, Via A. Manzoni 56, 20089 Rozzano, Italy; 5 Current address: BIOMETRA Department, University of Milan, Via Festa del Perdono, 7, 20122 Milan, Italy; 6 Current address: Department of Urology, University of Florida, 1600 SW Archer Road, Gainesville, FL 32610-0247, USA

## Abstract

**Introduction:**

Our recent data showed that signal transducers and activators of transcription 1 (STAT1), adenosine deaminase acting on RNA (ADAR), C-C motif chemokine ligand 2 (CCL2), and C-X-C motif chemokine 10 (CXCL10) were significantly elevated in a systemic lupus erythematosus (SLE) cohort compared to healthy donors. High and low STAT1 subsets were identified in SLE patient visits. The present study analyzed the correlation of common treatments used in SLE with the levels of these biomarkers.

**Methods:**

Peripheral blood leukocytes were collected from 65 healthy donors and 103 SLE patients, of whom 60 had samples from two or more visits. Total RNA was isolated and analyzed for the expression of mRNA and microRNA using Taqman real-time polymerase chain reaction (PCR) assays. Relative expression of interferon signature genes, CCL2, and CXCL10 were determined by the ^ΔΔ^CT method. Results were correlated with therapy using prednisone, mycophenolate mofetil, and hydroxychloroquine and analyzed by Wilcoxon/Kruskal-Wallis test and Fisher’s exact test.

**Results:**

CCL2 and CXCL10 were significantly higher in untreated patients compared to treated patients, however, in high STAT1 patient visits there is no significant difference between treated and untreated patients’ visits. When comparing linear regression fits of interferon (IFN) score with CCL2 and CXCL10, untreated patients and high STAT1 patients displayed significantly higher slopes compared to treated patients. There was no significant difference between the slopes of high STAT1 and untreated patients indicating that CCL2 and CXCL10 were correlated with type-I IFN in high STAT1 patients similar to that in untreated patients. CCL2 and CXCL10 levels in the high STAT1 subset remained high in treated patient visits compared to those of the low STAT1 subset.

**Conclusions:**

Among the biomarkers analyzed, only CCL2 and CXCL10 showed significantly reduced levels in treated compared to untreated SLE patients. STAT1, CCL2, and CXCL10 are potentially useful indicators of therapeutic action in SLE patients. Further work is needed to determine whether high STAT1 levels convey resistance to therapies commonly used to treat SLE and whether STAT1 inhibitors may have therapeutic implication for these patients.

## Introduction

Systemic lupus erythematosus (SLE) is a systemic autoimmune rheumatic disease affecting multiple systems and organs in the body. Several genetic and environmental factors have been implicated in SLE etiopathogenesis. Even though type I interferon (IFN-I: IFNα and IFNβ) was identified 30 years ago to be elevated in SLE patient serum, it is only in recent years that its increased expression has been rediscovered and postulated to play a key role in disease pathogenesis in the majority of patients [1-4].

In addition to IFN-I, STAT1 (signal transducers and activators of transcription 1), an interferon-inducible gene, is involved in type I, II, and III IFN signaling and is reported to be upregulated in SLE [5]. Besides STAT1, interferon-regulated chemokines also play a role in SLE pathogenesis [6]. C-C motif chemokine ligand 2 (CCL2) and C-X-C motif chemokine 10 (CXCL10) have been implicated in SLE as good indicators of potential flares [7]. The role of CCL2 in diseases such as psoriasis, rheumatoid arthritis, and multiple sclerosis has incited additional interest on its role in SLE [8]. Both CCL2 and CXCL10 depend upon the Jak/STAT pathway activation for induction by interferon [9-11] and these two chemokines were identified as one of the 12 upregulated proteins in SLE [6].

The role of microRNAs (miRNAs) has also been implicated in autoimmunity [12,13]. miR-146a was reported to be underexpressed in peripheral blood mononuclear cells of Chinese SLE patients [14]. The function of miR-146a is now known to regulate innate immune response and endotoxin tolerance [15-18]. miR-146a has also been reported to be overexpressed in Sjögren’s syndrome [19], psoriasis [20,21], and rheumatoid arthritis [22-24].

In an accompanying manuscript, we described high and low STAT1 populations in SLE patients [25]. In the low STAT1 population, levels of STAT1 correlated well with IFN score; however, in the high STAT1 population they did not. More importantly, high STAT1 patients displayed elevated expression of CCL2 and CXCL10, but no significant differences were observed for IFN score and tumor necrosis factor alpha (TNFα) between high and low STAT1. Finally, when the slope of the linear regression representing the rate of change of CCL2 or CXCL10 per unit of change of IFN score was analyzed, the slopes of CCL2/IFN score and CXCL10/IFN score were significantly greater in the high STAT1 patients compared to the low STAT1 patients indicating that STAT1 potentially enhanced CCL2 and CXCL10 response to IFN-I [25].

The current therapies for SLE primarily aim to suppress the inflammation and autoimmune response. Commonly used therapies include prednisone (PDN), mycophenolate mofetil (MMF), and hydroxychloroquine (HCQ). PDN is a synthetic glucocorticoid that suppresses inflammation by inhibiting nuclear factor kappa B (NF-кB). It inhibits monocyte and neutrophil inflammatory functions as well as B and T cell responses [26]. Synthetic glucocorticoid, such as dexamethasone and PDN can inhibit phosphorylation of STAT1 and potentially blocks IFN induction by suppressing INF receptor (IFNAR) signaling [27]; however, it has been shown that dexamethasone also upregulates STAT1 transcription [27]. This inhibition of STAT1 function while increasing its transcription appears to be counterintuitive but may represent a case of cell adapting to compensate for the loss of functional STAT1. Increases in STAT1 levels may lead to undesired consequences [28]. MMF is a cytotoxic drug commonly used to prevent organ rejection after transplantation and also to treat autoimmune diseases such as SLE. MMF is a reversible inhibitor of inosine monophosphate dehydrogenase that blocks the *de novo* synthesis of guanosine nucleotides [29]. The latter is required for growth and proliferation of T and B cells, as they lack the scavenger pathway and are unable to compensate for the inhibition of *de novo* synthesis of guanosine. Inhibition of T and B cell growth blocks autoimmune response and leads to decrease in autoantibody production and T-cell-mediated tissue damage. The antimalarial drug HCQ functions by increasing the pH of endosomal vesicles. This disrupts antigen processing and inhibiting toll-like receptor (TLR) 3, 7, 8, and 9 activity [29-31]; furthermore, HCQ can inhibit macrophage production of interleukin-1 and interleukin-6 [29]. Since TLR7/9 have been implicated in inciting IFN-I production due to recognition of self RNA/DNA, the blockade of these TLRs could be attenuating IFN-I production and antigen processing for presentation of T cells by antigen-presenting cells such as dendritic cells.

In this study, we analyze differences in the expression of various biomarkers, including STAT1, ADAR, CCL2, CXCL10, and miR-146a, in SLE patients treated with PDN, MMF, and HCQ versus untreated and healthy donors.

## Methods

### Healthy donors and SLE patients

Patient information is as described in the accompanying manuscript [25]. In brief, whole blood was collected from a total of 103 SLE patients and 65 healthy donors enrolled in the University of Florida Center for Autoimmune Diseases registry from 2008 to 2011. Healthy donors were selected based on no history of autoimmune disease, while all SLE patients satisfied the American College of Rheumatology criteria [32]. There were a total of 180 SLE visits with sequential samples collected in 60 SLE patients [25]. Healthy donors only visited the clinic once; therefore, they represent a single visit. Among the total of 180 visits, SLE patients were active in 49 visits according to the SLE disease activity index (SLEDAI) score >4. All human blood samples were obtained from enrolled individuals with the approval of institutional review board at the University of Florida. This study meets and is in compliance with all ethical standards in medicine and informed consent was obtained from all patients according to the Declaration of Helsinki.

### Data collection

RNA samples were isolated from peripheral blood leukocytes for each patient visit and analyzed for gene expression using TaqMan real-time PCR assays as described in the accompanying manuscript [25]. Anti-double-stranded DNA (dsDNA) levels, C3 and C4 complement levels, IFN score, and SLEDAI score were obtained as described [25]. C3 and C4 below 90 and 15 mg/dl, respectively, are considered subnormal levels.

### Data analysis

TaqMan real-time PCR assays were used to measure gene expression. The copy number of miR-146a was normalized to total loaded RNA whereas mRNA levels were normalized to 18S RNA. Copy number of miR-146a was determined using a standard curve with synthetic miR-146a (Integrated DNA Technologies Inc., Coralville, IA, USA) [33]. Relative expression of mRNA was determined by the ^ΔΔ^C_T_ method [34]. SLE patients were primarily treated with PDN, MMF, and/or HCQ. Correlations of all therapies during each patient visit were analyzed with levels of different SLE biomarkers. No patient in our SLE cohort was treated with belimumab, a B-cell-activating factor (BAFF) inhibitor approved by the FDA for SLE [35].

Analyses were performed using SAS version 9.2 and JMP Genomics version 5 (SAS, Cary, NC, USA). Wilcoxon/Kruskal-Wallis test was used to evaluate statistical significance between groups. Fisher’s exact test was used to examine the contingency between SLEDAI and therapy. Normal distribution of IFN score, CCL2, and CXCL10 as well as the bimodal distribution of STAT1 in SLE patients and healthy donor (HD) visits was identified as described in the accompanying manuscript [25]. Spearman Rho (ρ) coefficient was used to determine monotonic associations in the study. Coefficient of determination (r^2^) was used to determine linear correlations. Significance between slopes was evaluated by analysis of covariance (ANCOVA). *P* values less than 0.05 were considered significant. The Generalized Estimating Equation (GEE) model for repeated measures was used to account for possible within subject effects from patients with multiple visits [36].

## Results

### Comparison in the levels of various biomarkers in SLE patient visits with or without treatment

**Figure 1:**
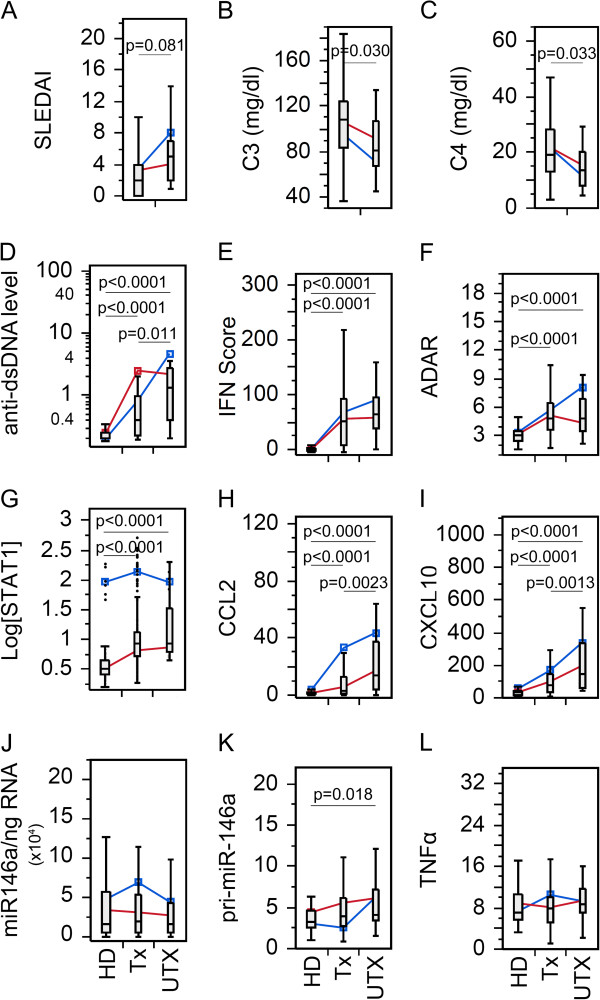
**Comparison in the levels of various clinical parameters and biomarkers in SLE patient visits with or without treatment. (A)** Disease activity, **(B-C)** complement levels, **(D)** anti-dsDNA antibody levels, **(E)** IFN score, **(F)** ADAR, **(G)** STAT1, **(H)** CCL2, **(I)** CXCL10, **(J)** miR-146a, **(K)** pri-miR-146a, and **(L)**. TNFα in treated (Tx) and untreated (UTX) SLE patient visits as well as healthy donors (HD). Data are presented as box plot. All groups were compared among each other and only significant *P* values are shown indicating each specific comparison. Average trend lines for high STAT1 (blue) and low STAT1 (red) patient visit subsets are also shown for comparison. Detail comparison between high and low STAT1 subsets are shown in Additional file 1: Figure S2. ADAR, adenosine deaminase acting on RNA; CCL2, C-C motif chemokine ligand 2; CXCL10, C-X-C motif chemokine 10; dsDNA, double-stranded DNA; IFN, interferon; SLE, systemic lupus erythematosus; STAT1, signal transducer and activator of transcription 1; TNFα, tumor necrosis factor alpha.

Changes in C3, C4, and anti-dsDNA antibody levels in SLE patient visits, and mRNA expression levels of various biomarkers in peripheral blood leucocytes were examined for possible effects of therapy (Figure 1). As expected, SLEDAI (Figure 1A) and anti-dsDNA autoantibody (Figure 1D) levels were significantly lower in treated (Tx) than untreated (UTX) patients, while C3 (Figure 1B) and C4 (Figure 1C) were significantly higher in Tx than UTX patients. Overall, anti-dsDNA autoantibody, IFN scores, adenosine deaminase acting on RNA (ADAR), STAT1, CCL2, and CXCL10, were significantly lower in HD than either UTX or Tx SLE patient visits (Figure 1D-I). However, there were no significant differences among the groups for miR-146a (Figure 1J) and TNFα (Figure 1L) expression. pri-miR-146a showed significantly higher level only in UTX compared to HD.

Bimodal distribution of STAT1 in SLE patient and HD visits was identified as described in the accompanying manuscript [25]. To further elucidate the influence of high and low STAT1 populations, UTX and HD from Figure 1 were further examined by comparing the high (blue) and low (red) STAT1 groups (See Additional file 1: Figure S1). As expected, regardless of STAT1 levels, UTX was significantly higher in anti-dsDNA, IFN score, ADAR, CCL2, and CXCL10 than HD (See Additional file 1: Figure S1A-C,E,F) while there was no difference in STAT1, miR-146a, pri-miR-146a, and TNFα (See Additional file 1: Figure S1D, G-I). High STAT1 HD displayed higher levels of STAT1, CCL2, and CXCL10 (See Additional file 1: Figure S1D-F) than low STAT1 HD; however, for the remaining biomarkers, there were no significant differences. Levels of various biomarkers in UTX patient visits were not significantly different by STAT1 levels with the exception of STAT1 (See Additional file 1: Figure S1). Due to the lack of significant difference in levels of biomarkers between high and low STAT1 UTX patients, UTX were not separated in any subsequent analysis.

Next, various biomarker levels in treated patients with high versus low STAT1 visits were compared with UTX and HD. Overall two very important outcomes became apparent. First, the lack of significant difference between UTX and high STAT1 for SLEDAI, IFN score, ADAR, CCL2, and CXCL10 (See Additional file 1: Figure S2A-F,H,I) potentially indicating that the pathology of high STAT1 Tx patients resembled that of UTX patients. Second, high STAT1 Tx patient visits displayed significantly higher CCL2 and CXCL10 (See Additional file 1: Figure S2H,I) than the low STAT group, which might be indicators of increased pathological activity. miR-146a also showed the same trend, however, high STAT1 Tx patients have higher levels of miR-146a than UTX (See Additional file 1: Figure S2J). Interestingly, pri-miR-146a appeared to have an opposite trend (See Additional file 1: Figure S2K).

### Comparison of individual therapies

**Figure 2:**
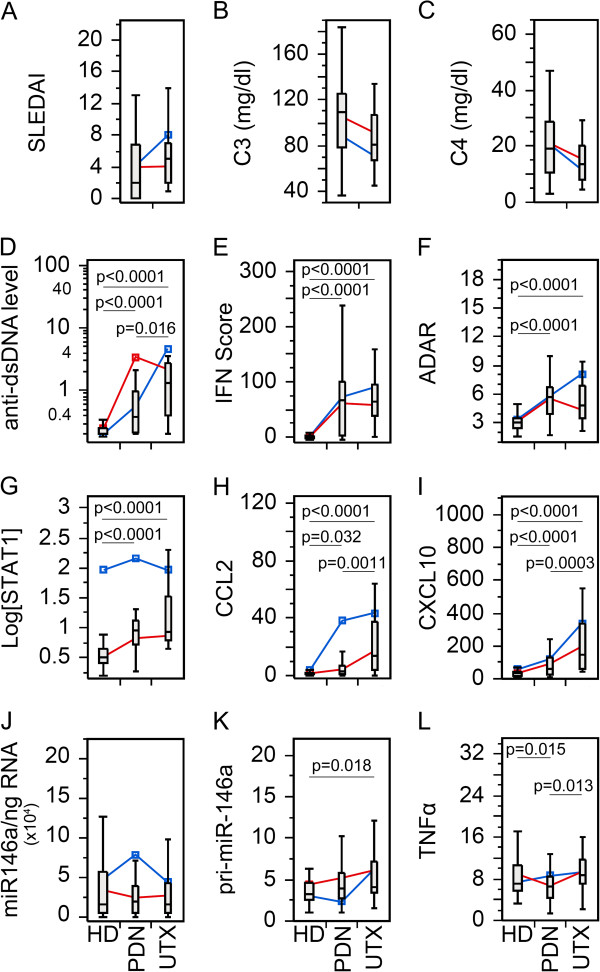
**Comparison of the levels of various biomarkers in the SLE patient visits with prednisone (PDN) therapy versus untreated.** Data were analyzed as in Figure 1 except only patients receiving PDN in the treated patient population were included. **(A)** Disease activity, **(B-C)** complement levels, **(D)** anti-dsDNA antibody levels, **(E)** IFN score, **(F)** ADAR, **(G)** STAT1, **(H)** CCL2, **(I)** CXCL10, **(J)** miR-146a, **(K)** pri-miR-146a, and **(L)** TNFα in treated (Tx) and untreated (UTX) SLE patient visits as well as healthy donors (HD). Data are presented as box plot. All groups were compared among each other and only significant *P* values are shown indicating each specific comparison. Average trend lines for high STAT1 (blue) and low STAT1 (red) patient visit subsets are also shown for comparison. Detail comparison between high and low STAT1 subsets are shown in Additional file 1: Figure S3. ADAR, adenosine deaminase acting on RNA; CCL2, C-C motif chemokine ligand 2; CXCL10, C-X-C motif chemokine 10; dsDNA, double-stranded DNA; IFN, interferon; SLE, systemic lupus erythematosus; STAT1, signal transducer and activator of transcription 1; TNFα, tumor necrosis factor alpha.

**Figure 3:**
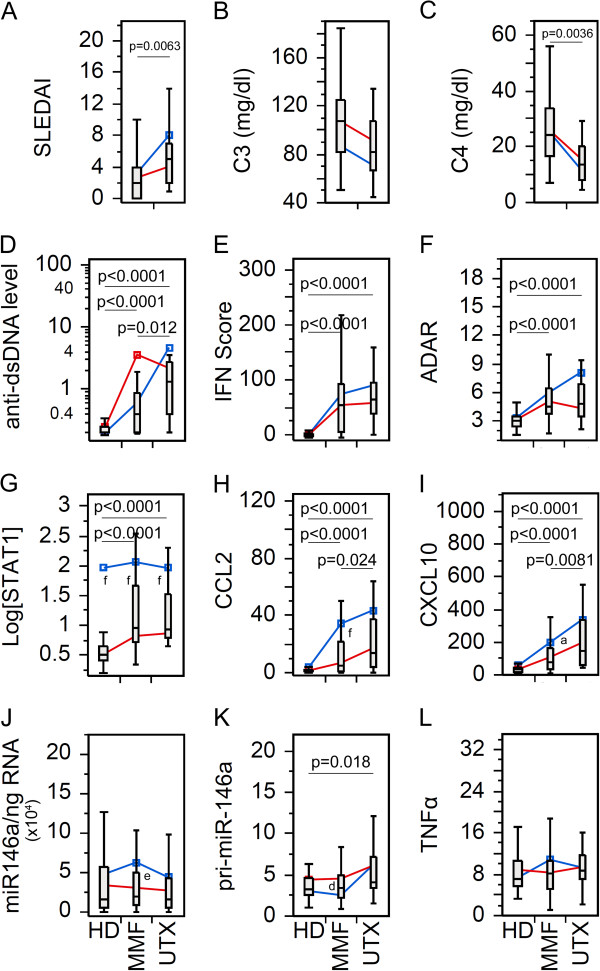
**Comparison of the levels of various biomarkers in the SLE patients visits with hydroxychloroquine (HCQ) therapy versus untreated.** Data were analyzed as in Figure 1 except only patients receiving HCQ in the treated patient population were included. **(A)** Disease activity, **(B-C)** complement levels, **(D)** anti-dsDNA antibody levels, **(E)** IFN score, **(F)** ADAR, **(G)** STAT1, **(H)** CCL2, **(I)** CXCL10, **(J)** miR-146a, **(K)** pri-miR-146a, and **(L)** TNFα in treated (Tx) and untreated (UTX) SLE patient visits as well as healthy donors (HD). Data are presented as box plot. All groups were compared among each other and only significant *P* values are shown indicating each specific comparison. Average trend lines for high STAT1 (blue) and low STAT1 (red) patient visit subsets are also shown for comparison. Detail comparison between high and low STAT1 subsets are shown in Additional file 1: Figure S4. ADAR, adenosine deaminase acting on RNA; CCL2, C-C motif chemokine ligand 2; CXCL10, C-X-C motif chemokine 10; dsDNA, double-stranded DNA; IFN, interferon; SLE, systemic lupus erythematosus; STAT1, signal transducer and activator of transcription 1; TNFα, tumor necrosis factor alpha.

**Figure 4:**
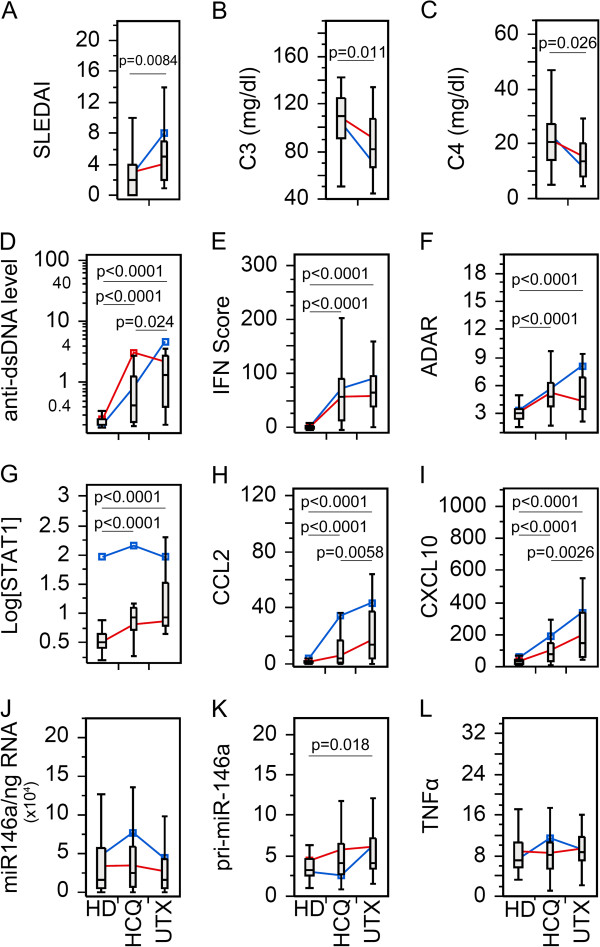
**Comparison of the levels of various biomarkers in the SLE patients visits with mycophenolate mofetil (MMF) therapy versus untreated.** Data were analyzed as in Figure 1 except only patients receiving MMF in the treated patient population were included. **(A)** Disease activity, **(B-C)** complement levels, **(D)** anti-dsDNA antibody levels, **(E)** IFN Score, **(F)** ADAR, **(G)** STAT1, **(H)** CCL2, **(I)** CXCL10, **(J)** miR-146a, **(K)** pri-miR-146a, and **(L)** TNFα in treated (Tx) and untreated (UTX) SLE patient visits as well as healthy donors (HD). Data are presented as box plot. All groups were compared among each other and only significant *P* values are shown indicating each specific comparison. Average trend lines for high STAT1 (blue) and low STAT1 (red) patient visit subsets are also shown for comparison. Detail comparison between high and low STAT1 subsets are shown in Additional file 1: Figure S5. ADAR, adenosine deaminase acting on RNA; CCL2, C-C motif chemokine ligand 2; CXCL10, C-X-C motif chemokine 10; dsDNA, double-stranded DNA; IFN, interferon; SLE, systemic lupus erythematosus; STAT1, signal transducer and activator of transcription 1; TNFα, tumor necrosis factor alpha.

Since many patients were on more than one medication, we wanted to compare biomarkers in patients with an individual drug. As for PDN (Figure 2), by excluding patients not receiving PDN from the Tx group, there was no statistical significant difference between PDN Tx and UTX with SLEDAI, C3, and C4 (Figure 2A-C). However, SLE patients receiving PDN were more frequently inactive (*P* = 0.0071; likelihood ratio: 7.44) than active by SLEDAI score. The remaining biomarkers (Figure 2D-L) showed similar significant trends as seen in the Tx population (Figure 1D-L), which might indicate that the overall results were from a combinatory effect of the therapy and/or all therapy had similar effects on these biomarkers. To appreciate these results, HCQ and MMF were also analyzed in the same manner (Figures 3 and 4). SLEDAI, C3, and C4 were significantly different between HCQ patients and UTX (Figure 3A-C); however, only SLEDAI and C4 were significantly different between MMF and UTX patient visits (Figure 4A-C). The results for SLEDAI were consistent with SLE patient visits treated with HCQ (*P* = 0.0002; likelihood ratio: 13.9) or with MMF (*P* <0.0001; likelihood ratio: 16.1) were more likely to be in inactive states. The remaining biomarkers for HCQ (Figure 3D-L) and MMF (Figure 4D-L) resembled those in the entire Tx population (Figure 1D-L).

After establishing the basic role of high and low STAT1, their correlation was further explored for each therapy. Beginning with PDN, TNFα was significantly decreased in the low STAT1 PDN patient visits relative to UTX and HD; however, high STAT1 PDN patient visits were not significantly different (See Additional file 1: Figure S3L). This trend was not observed for either HCQ or MMF patients (See Additional file 1: Figure S4L, S5L). High and low STAT1 patients under PDN therapy (See Additional file 1: Figure S3A-C) did not display any significant differences for SLEDAI, C3, and C4, which resembled the earlier results (Figure 2A-C). This differed for HCQ and MMF where low STAT1 patient visits were significantly lower than UTX patient visits for SLEDAI, and higher in C3 and C4 (See Additional file 1: Figure S4A-C, 5A-C). In PDN, HCQ, and MMF patient visits, CCL2 and CXCL10 was significantly elevated in the high STAT1 population compared to the low STAT1, but significantly different from UTX (See Additional file 1: Figures S3H,I; S4H,I; S5H,I). This resembled what was observed earlier in high/low STAT1 Tx patients (See Additional file 1: Figure S2H,I) suggesting that high STAT1 patients might maintain high levels of CCL2 and CXCL10 regardless of the therapy used.

The relationship between miR-146a and pri-miR-146 was particularly revealing when the analyses took into account the difference in high STAT1 versus low STAT1 status. While miR-146a did not show any significant difference in PDN, HCQ, and MMF patient visits (Figures 2J, 3J and 4J), high versus low STAT1 Tx patient visits (See Additional file 1: Figure S2J) as well as patients treated with PDN, HCQ, and MMF (See Additional file 1: Figure S3J, S4J, S5J) revealed that high STAT1 patient visits were significantly higher in miR-146a than low STAT1 patient visits, UTX, and HD. In contrast, pri-miR-146a levels were significantly lower in high STAT1 patient visits than in low STAT1 patient visits, UTX, and HD for high/low STAT1 Tx patient visits (See Additional file 1: Figure S2K) as well as patients treated with PDN, HCQ, and MMF (See Additional file 1: Figures S3K, S4K, S5K). The reverse trend seen between pri-miR-146a and miR-146a was probably due to differences in conversion from primary to mature miRNA or potential differences in their intrinsic stability.

**Figure 5:**
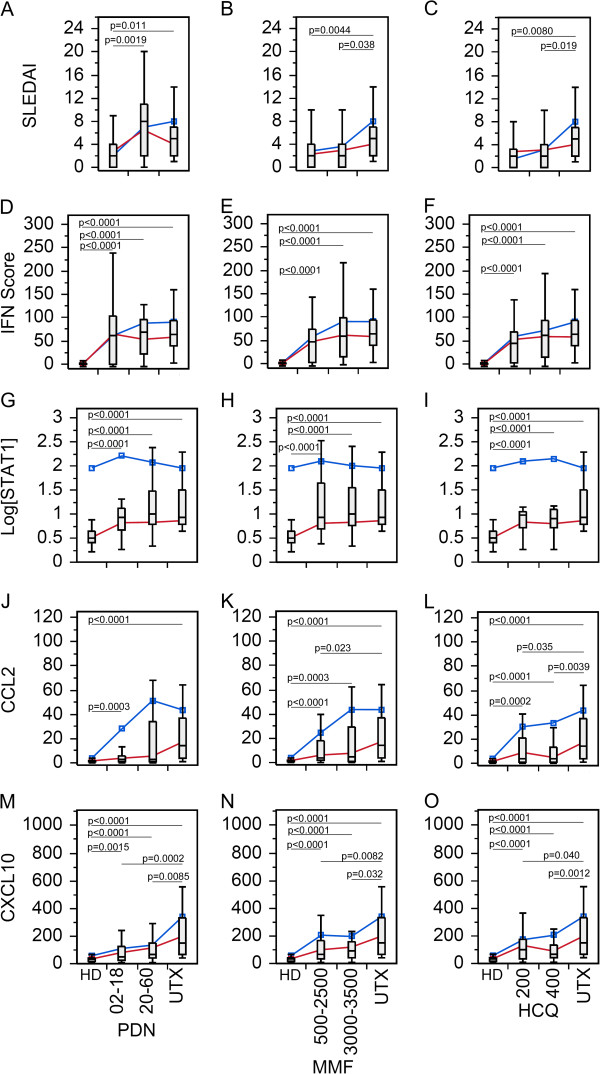
**Comparison of the levels of various biomarkers in the SLE patient visits given PDN, MMF, and HCQ therapy at high or low dosage.** Differences between doses of PDN **(D, G, J, M)**, MMF **(B, E, H, K, N)**, and HCQ **(C, F, I, L, O)** were not significant with the exception of SLEDAI **(A)**, and in fact SLEDAI scores were higher for PDN dose of 20 to 60 mg/day compared to the 2 to 18 mg/day of dose. HCQ, hydroxychloroquine; MMF, mycophenolate mofetil; PDN, prednisone; SLE, systemic lupus erythematosus; SLEDAI, SLE disease activity index.

Therapy dosage could vary based on disease manifestation and severity. To examine the effects of therapy dosage, the PDN, MMF, and HCQ treated patients were separated by dosage (Figure 5). As dosage increased so did the levels of the biomarkers that are supposed to correlate with disease activity. This might be attributed to the way therapy was administered. As the disease activity of patients became higher, prescription of higher doses of therapy might be expected. Essentially, therapy dosage might act as a marker of disease activity. Interestingly, the high STAT1 patient visits (blue) appeared to show higher levels of STAT1, CCL2 and CXCL10 than in low STAT1 patient visits as therapy dose increased (Figure 5, Additional file 1: Figure S6).

### Association between CCL2, IFN score, and therapy

The accumulated evidence so far appeared that patients with high levels of STAT1 were maintaining high CCL2 and CXCL10 expression even during therapy; we tested how STAT1 levels affected the association of CCL2 and CXCL10 with IFN score. Since CCL2 and CXCL10 are known to be induced by interferon, this would suggest a positive covariation where CCL2 and CXCL10 increase as IFN score increases. The slope of CCL2/IFN score and CXCL10/IFN score thus represents the association between CCL2 and CXCL10 with IFN score. By comparing the slope between groups, the effects of therapy on the association of CCL2 and CXCL10 with IFN score could be examined. For example, when the slope of CCL2/IFN score was greater for UTX than that of a particular therapy, it suggested that the decreased association in CCL2/IFN score for the treated patients was a result of that particular therapy or due to other conditions of the patients.

**Figure 6:**
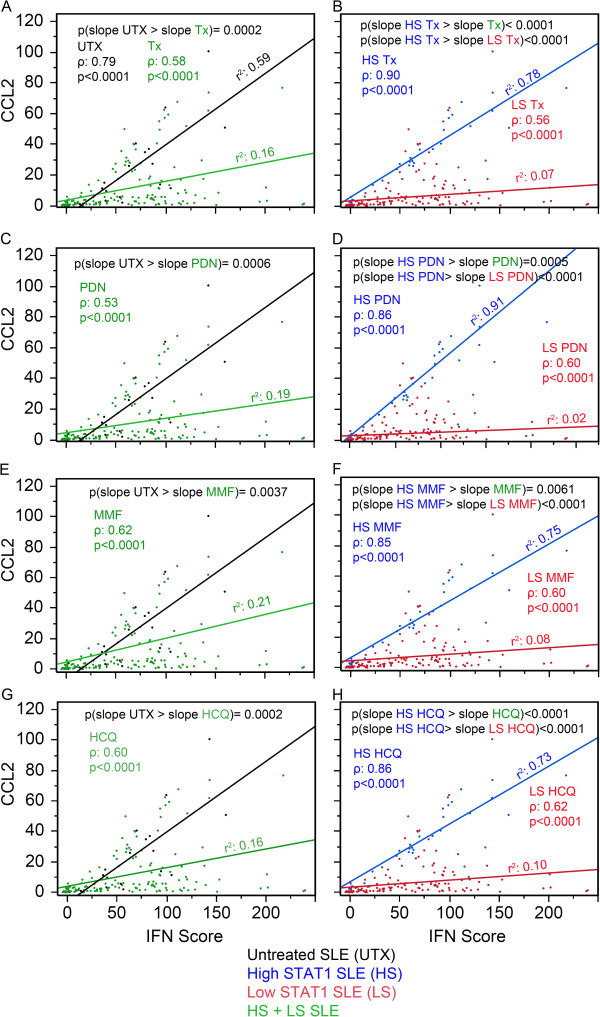
**Association between CCL2, IFN score, and therapy. (A)** The relationship of CCL2 versus IFN score presented as a slope was analyzed in untreated (UTX, black) and treated SLE patient visits (Tx, green). Similar analyses were carried out for PDN-treated **(C)**, MMF-treated **(E)**, and HCQ-treated patient visits **(G)** as well as for high STAT1 (blue) and low STAT1 (red) for Tx **(B)**, PDN-treated **(D)**, MMF-treated **(F)**, and HCQ-treated patient visits **(H)**. CCL2, C-C motif chemokine ligand 2; HCQ, hydroxychloroquine; IFN, interferon; MMF, mycophenolate mofetil; PDN, prednisone; SLE, systemic lupus erythematosus; SLEDAI, SLE disease activity index; STAT1, signal transducer and activator of transcription 1.

**Figure 7:**
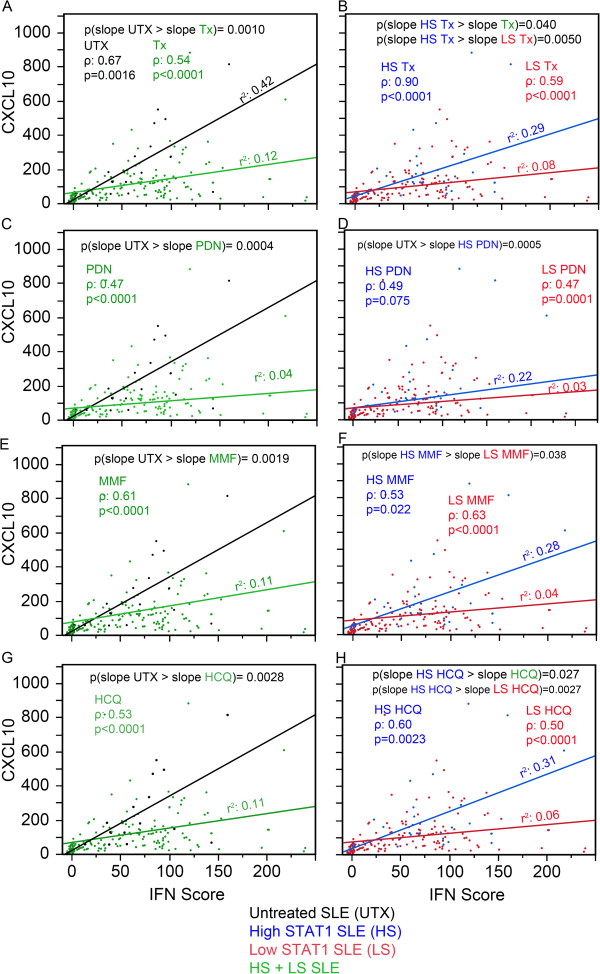
**Association between CXCL10, IFN score, and therapy.** Data were analyzed as in Figure 6 except that CCL2 was substituted by CXCL10. **(A)** The relationship of CCL2 versus IFN score presented as a slope was analyzed in untreated (UTX, black) and treated SLE patient visits (Tx, green). Similar analyses were carried out for PDN-treated **(C)**, MMF-treated **(E)**, and HCQ-treated patient visits **(G)** as well as for high STAT1 (blue) and low STAT1 (red) for Tx **(B)**, PDN-treated **(D)**, MMF-treated **(F)**, and HCQ-treated patient visits **(H)**. CCL2, C-C motif chemokine ligand 2; CXCL10, C-X-C motif chemokine 10; HCQ, hydroxychloroquine; IFN, interferon; MMF, mycophenolate mofetil; PDN, prednisone; SLE, systemic lupus erythematosus; SLEDAI, SLE disease activity index; STAT1, signal transducer and activator of transcription 1.

When the association of CCL2 with IFN score was plotted as shown in Figure 6A, three items were noted. First, both UTX and Tx were monotonic and increased as observed from the Spearman rho coefficient (ρ). Second, both UTX and Tx displayed a linear component as described by the coefficient of determination (r^2^) and UTX had a greater linearity than Tx. Third, UTX had a significantly greater slope for CCL2/IFN score than Tx (*P* = 0.0002, black versus green line) potentially indicating that therapy decreased CCL2 responsiveness to IFN-I. In Figure 6B, Tx was segregated into high and low STAT1. Similarly, high STAT1 Tx and low STAT1 Tx were monotonic, increasing and linear. High STAT1 Tx displayed a significantly higher slope than low STAT1 Tx (Figure 6B, *P* <0.0001, blue versus red line) and significantly higher slope than Tx (Figure 6A-B, *P* <0.0001, blue versus green line) indicating that CCL2 responsiveness to IFN-I in high STAT1 patients was more similar to that of the UTX patients. Overall similar results were observed for PDN, MMF, and HCQ (Figure 6C-H). The same analysis was performed for CXCL10 (Figure 7). The results were similar to those of CCL2 (Figure 6) with the exception for PDN and MMF in the high versus low STAT1 patient visits (Figure 7D,F). For PDN, high STAT1 patient visits were not significantly different than low STAT1 (blue versus red line); in addition, high STAT1 PDN was significantly lower than UTX (Figure 7C-D, *P* = 0.0005, blue versus black line) and this might indicate that PDN affected CXCL10 response to IFN-1. For MMF, high STAT1 patient visits had significantly higher slope than low STAT1 patient visits (Figure 7F, *P* = 0.038, blue versus red line); however, high STAT1 MMF was not significantly different in CXCL10 from MMF-treated patient visits (Figure 7E-F, blue versus green line).

### Expression of CCL2 and CXCL10 in high versus low STAT1 patient subsets with individual and combined therapy

**Figure 8:**
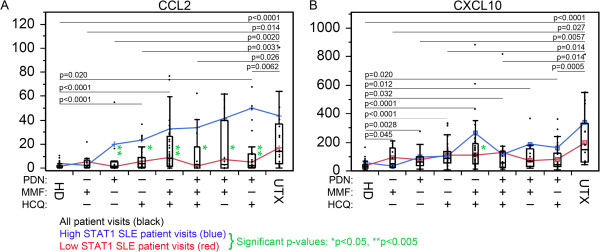
**The expression of CCL2 and CXCL10 in high versus low STAT1 patient subsets with individual and combined therapy. (A)** CCL2 levels in HD (healthy donor), untreated (UTX), and other patient visits under treatment with different combinations of PDN, MMF, and HCQ were plotted (black bars). Only significant differences comparing each treatment group to either HD or UTX are indicated as black lines with *P* value shown. Data segregating into high STAT1 (blue line) and low STAT1 (red line) subsets are also shown and significant differences for these subsets are indicated by green asterisks. **(B)** CXCL10 data were analyzed similarly. CCL2, C-C motif chemokine ligand 2; CXCL10, C-X-C motif chemokine 10; HCQ, hydroxychloroquine; HD, healthy donor; IFN, interferon; MMF, mycophenolate mofetil; PDN, prednisone; SLE, systemic lupus erythematosus; SLEDAI, SLE disease activity index; STAT1, signal transducer and activator of transcription 1.

Finally, all possible therapy combinations (MMF, PDN, HCQ, HCQ + MMF, PDN + MMF + HCQ, PDN + MMF, PDN + HCQ, and UTX) were compared for the expression of all biomarkers. Interestingly, while there was no significant differences in IFN score, STAT1, ADAR, pri-miR-146a, and mature miR-146a observed between UTX and the various treatments (data not shown), CCL2 and CXCL10 displayed significant trends (Figure 8). For nearly every treatment, CCL2 was decreased compared to UTX (Figure 8A). Overall significant decrease in CCL2 transcripts in those treated compared to UTX patient visits indicated that therapy was affecting CCL2 transcription; however, this might not be true for high STAT1 patient visits (blue line) as they were significantly higher in CCL2 than the low STAT1 patients (red line) for nearly every treatment (Figure 8A). The low STAT1 patients appeared to be responsive to therapy as they were significantly lower than UTX and the majority was not significantly different from HD (See Additional file 1: Figure S7A). This was reversed in the high STAT1 patients where HD were significantly lower than treated patients and the majority were not significantly different from UTX patients (See Additional file 1: Figure S7b).

The results for CXCL10 were not as consistent as CCL2. UTX patients were significantly higher in CXCL10 than any treated groups (Figure 8B). Both the treated patient visits, high STAT1 patient visits, and the majority of low STAT1 patient visits were significantly lower than UTX (Figure 8A, Additional file 1: Figure S8). While the low STAT1 patient visits were significantly lower in CXCL10 than UTX, the high STAT1 were not significantly different from UTX (See Additional file 1: Figure S8) potentially again supporting that high STAT1 levels contribute to maintain the high level of CXCL10 in patients under therapy.

## Discussion

Our study focused on the difference in the levels of SLE biomarkers and their relationship with interferon, CCL2, and CXCL10 in SLE patients given different therapy. IFN-I and interferon signature genes were reported to be elevated both at the mRNA level based on data from microarray analyses and even at the protein level in the serum of SLE patients [4,37-39]. Not surprisingly, our results reaffirm the elevated expression of ADAR, STAT1, CCL2, and CXCL10 in SLE patients [25] as reported in the literature [1,2,6,7,37,40].

CCL2 and CXCL10 levels are lower in treated versus untreated SLE patients. The majority of SLE patient visits were receiving therapy at the time of sample collection. SLE patient visits using PDN, MMF, and HCQ as well as therapy combinations displayed no significant decrease of IFN score, STAT1, ADAR, pri-miR-146a, and mature miR-146a compared to untreated. Linear regression analyses treating the patient visits as independent variables (in Figures 6 and 7) yielded essentially the same conclusion when compared to using the GEE model for repeated measures (data not shown).

PDN is a glucocorticoid that suppresses NF-кB signaling [41]. It is unclear how or even if PDN suppresses IFN production. Glucocorticoids have been reported to suppress STAT1 phosphorylation (pSTAT1) [27], but depending upon cell type and profile, they can also lead to changes in the transcription of STAT1 [28,42]. STAT1 is important for CCL2 and CXCL10 induction by INF [43-45]. Furthermore, the decrease in pSTAT1 could explain why CCL2 and CXCL10 decreased in the low STAT1 patients. The increase in STAT1 expression may be an attempt to compensate for decreased pSTAT1 levels and may possibly explain the occurrence of the high STAT1 patients. This may also be the reason for CCL2 and CXCL10 increase in high STAT1 patients and why CCL2 and CXCL10 are not as significantly lower in SLE patients undergoing therapy in the high STAT1 patients compared to the low STAT1 patients.

On the other hand, CCL2 and CXCL10 expression levels in SLE patients undergoing therapy were significant lower than untreated patients. PDN has been previously reported to decrease CCL2 and CXCL10 expression [46-48]. If PDN reduces pSTAT1 levels, this may explain in part the decrease of CCL2 and CXCL10 expression due to the role of STAT1 in chemokine signaling [43-45]. In high STAT1 SLE patients, CCL2 and CXCL10 did not significantly change from untreated SLE patients, possibly indicating that the elevated levels of STAT1 are facilitating a pathogenic pattern occurring in the untreated patients. In part, STAT1 may be increasing to compensate for inhibition of STAT1 phosphorylation and maintain CCL2 and CXCL10 levels as in the untreated patients. STAT1 has been associated with therapy resistance in cancer. STAT1 overexpression protects cancers from DNA-damaging agents including radiation therapies and chemotherapies in different cancer types [49]. Radioresistant nu61 derived from radiosensitive SCC61 tumors displayed 49 overexpressed genes; of these 49 genes, 31 were ISGs also including STAT1 [50]. Furthermore when STAT1 was overexpressed in SCC61 cells, it displayed radioresistance [51]. Similarly, human fibroblasts repeatedly exposed to IFN-I displayed radio-resistance [52]. In 10 cancer cell lines, STAT1 expression correlated with resistance to doxorubicin and topoisomerase-II inhibitors [53]. In addition, 14 ovarian cancer lines were observed for resistance to platinum compounds where STAT1 was associated with resistance to cisplatin and AMD473 [54]. These associations between therapy resistance and STAT1 in cancer may explain the association of STAT1 levels with higher CCL2 and CXCL10 and the apparent lack of therapy sensitivity in high STAT1 patients.

## Conclusions

Increases in CCL2 and CXCL10 have been associated with SLE patients entering a state of flare activity [6,7]. We consider reduction of CCL2 and CXCL10 as good indicators of successful therapy, while elevation in STAT1 levels may indicate therapy resistance. Further work is needed to determine the role that STAT1 plays in therapy, but this study gives insight to a potentially new role for STAT1 in SLE. Our study raises an interesting question whether SLE patients with high STAT1 status can benefit from therapy with specific STAT1 inhibitors [55].

## Abbreviations

ADAR: adenosine deaminase acting on RNA; CCL2: C-C motif chemokine ligand 2; CXCL10: C-X-C motif chemokine 10; dsDNA: double-stranded DNA; HCQ: hydroxychloroquine; HD: healthy donors; IFNAR: interferon receptor; IFN-I: type I interferon; miRNA: microRNA; MMF: mycophenolate mofetil; PDN: prednisone; pSTAT1: phosphorylation STAT1; SLE: systemic lupus erythematosus; SLEDAI: SLE disease activity index; STAT: signal transducers and activators of transcription; TLR: toll-like receptor; TNFα: tumor necrosis factor alpha; Tx: treated; UTX: untreated.

## Competing interests

The authors declare that they have no competing interests.

## Authors’ contributions

PRDG carried out the experiments. PRDG, MS and EKLC designed the study. PRDG, AC, and MS performed the statistical analysis. ESS, AC, and WHR enrolled patients for the study, collected information and maintained the database. PRDG, AC, and EKLC drafted the manuscript. All authors read and approved the final manuscript.

## Supplementary Material

Additional file 1: Figure S1 Expression of different biomarkers in high versus low STAT1 populations in both SLE and healthy donors. **Figure S2.** Comparison of high and low STAT1 subsets of all treated to untreated SLE patient visits. **Figure S3.** Comparison of high and low STAT1 subsets of PDN treated patient visits to untreated patient visits. **Figure S4.** Comparison of high and low STAT1 subsets of HCQ treated patient visits to untreated patient visits. **Figure S5.** Comparison of high and low STAT1 subsets of MMF treated patient visits to untreated patient visits. **Figure S6.** Comparison of high and low STAT1 and dosage subsets on expression levels of the various biomarkers in the SLE cohort. **Figure S7.** Separate analyses of high and low STAT1 effects on CCL2 expression in various combined therapies. **Figure S8.** Separate analyses of high and low STAT1 effects on CXCL10 expression in various combined therapies. Click here for file

## References

[B1] Baechler EC Batliwalla FM Karypis G Gaffney PM Ortmann WA Espe KJ Shark KB Grande WJ Hughes KM Kapur V Gregersen PK Behrens TW Interferon-inducible gene expression signature in peripheral blood cells of patients with severe lupus Proc Natl Acad Sci USA 2003 16 2610 2615 10.1073/pnas.0337679100 12604793 PMC151388

[B2] Bennett L Palucka AK Arce E Cantrell V Borvak J Banchereau J Pascual V Interferon and granulopoiesis signatures in systemic lupus erythematosus blood J Exp Med 2003 16 711 723 10.1084/jem.20021553 12642603 PMC2193846

[B3] Crow MK Interferon pathway activation in systemic lupus erythematosus Curr Rheumatol Rep 2005 16 463 468 10.1007/s11926-005-0053-4 16303107

[B4] Preble OT Black RJ Friedman RM Klippel JH Vilcek J Systemic lupus erythematosus: presence in human serum of an unusual acid-labile leukocyte interferon Science 1982 16 429 431 10.1126/science.6176024 6176024

[B5] Karonitsch T Feierl E Steiner CW Dalwigk K Korb A Binder N Rapp A Steiner G Scheinecker C Smolen J Aringer M Activation of the interferon-gamma signaling pathway in systemic lupus erythematosus peripheral blood mononuclear cells Arthritis Rheum 2009 16 1463 1471 10.1002/art.24449 19404947

[B6] Bauer JW Baechler EC Petri M Batliwalla FM Crawford D Ortmann WA Espe KJ Li W Patel DD Gregersen PK Behrens TW Elevated serum levels of interferon-regulated chemokines are biomarkers for active human systemic lupus erythematosus PLoS Med 2006 16 e491 10.1371/journal.pmed.0030491 17177599 PMC1702557

[B7] Bauer JW Petri M Batliwalla FM Koeuth T Wilson J Slattery C Panoskaltsis-Mortari A Gregersen PK Behrens TW Baechler EC Interferon-regulated chemokines as biomarkers of systemic lupus erythematosus disease activity: a validation study Arthritis Rheum 2009 16 3098 3107 10.1002/art.24803 19790071 PMC2842939

[B8] Xia M Sui Z Recent developments in CCR2 antagonists Expert Opin Ther Pat 2009 16 295 303 10.1517/13543770902755129 19441905

[B9] Loetscher M Loetscher P Brass N Meese E Moser B Lymphocyte-specific chemokine receptor CXCR3: regulation, chemokine binding and gene localization Eur J Immunol 1998 16 3696 3705 10.1002/(SICI)1521-4141(199811)28:11<3696::AID-IMMU3696>3.0.CO;2-W 9842912

[B10] Weng Y Siciliano SJ Waldburger KE Sirotina-Meisher A Staruch MJ Daugherty BL Gould SL Springer MS DeMartino JA Binding and functional properties of recombinant and endogenous CXCR3 chemokine receptors J Biol Chem 1998 16 18288 18291 10.1074/jbc.273.29.18288 9660793

[B11] Han C Fu J Liu Z Huang H Luo L Yin Z Dipyrithione inhibits IFN-gamma-induced JAK/STAT1 signaling pathway activation and IP-10/CXCL10 expression in RAW264.7 cells Inflamm Res 2010 16 809 816 10.1007/s00011-010-0192-6 20372968 PMC7079753

[B12] Ceribelli A Satoh M Chan EKL MicroRNAs and autoimmunity Curr Opin Immunol 2012 16 686 691 10.1016/j.coi.2012.07.011 22902047 PMC3508200

[B13] Ceribelli A Yao B Dominguez-Gutierrez PR Nahid MA Satoh M Chan EKL MicroRNAs in systemic rheumatic diseases Arthritis Res Ther 2011 16 229 10.1186/ar3377 21787439 PMC3239341

[B14] Tang Y Luo X Cui H Ni X Yuan M Guo Y Huang X Zhou H de Vries N Tak PP Chen S Shen N MicroRNA-146A contributes to abnormal activation of the type I interferon pathway in human lupus by targeting the key signaling proteins Arthritis Rheum 2009 16 1065 1075 10.1002/art.24436 19333922

[B15] Taganov KD Boldin MP Chang KJ Baltimore D NF-kappaB-dependent induction of microRNA miR-146, an inhibitor targeted to signaling proteins of innate immune responses Proc Natl Acad Sci USA 2006 16 12481 12486 10.1073/pnas.0605298103 16885212 PMC1567904

[B16] Nahid MA Rivera M Lucas A Chan EK Kesavalu L Polymicrobial infection with periodontal pathogens specifically enhances microRNA miR-146a in ApoE−/− mice during experimental periodontal disease Infect Immun 2011 16 1597 1605 10.1128/IAI.01062-10 21263019 PMC3067556

[B17] Nahid MA Pauley KM Satoh M Chan EKL miR-146a is critical for endotoxin-induced tolerance: implication in innate immunity J Biol Chem 2009 16 34590 34599 10.1074/jbc.M109.056317 19840932 PMC2787321

[B18] Chan EKL Ceribelli A Satoh M MicroRNA-146a in autoimmunity and innate immune responses Ann Rheum Dis 2013 16 ii90-ii95 23253933 10.1136/annrheumdis-2012-202203 PMC7664460

[B19] Pauley KM Stewart CM Gauna AE Dupre LC Kuklani R Chan AL Pauley BA Reeves WH Chan EK Cha S Altered miR-146a expression in Sjogren’s syndrome and its functional role in innate immunity Eur J Immunol 2011 16 2029 2039 10.1002/eji.201040757 21469088 PMC3760391

[B20] Sonkoly E Stahle M Pivarcsi A MicroRNAs: novel regulators in skin inflammation Clin Exp Dermatol 2008 16 312 315 10.1111/j.1365-2230.2008.02804.x 18419608

[B21] Sonkoly E Wei T Janson PC Saaf A Lundeberg L Tengvall-Linder M Norstedt G Alenius H Homey B Scheynius A Stahle M Pivarcsi A MicroRNAs: novel regulators involved in the pathogenesis of psoriasis? PLoS One 2007 16 e610 10.1371/journal.pone.0000610 17622355 PMC1905940

[B22] Nakasa T Miyaki S Okubo A Hashimoto M Nishida K Ochi M Asahara H Expression of microRNA-146 in rheumatoid arthritis synovial tissue Arthritis Rheum 2008 16 1284 1292 10.1002/art.23429 18438844 PMC2749927

[B23] Pauley KM Satoh M Chan AL Bubb MR Reeves WH Chan EK Upregulated miR-146a expression in peripheral blood mononuclear cells from rheumatoid arthritis patients Arthritis Res Ther 2008 16 R101 10.1186/ar2493 18759964 PMC2575615

[B24] Stanczyk J Pedrioli DM Brentano F Sanchez-Pernaute O Kolling C Gay RE Detmar M Gay S Kyburz D Altered expression of MicroRNA in synovial fibroblasts and synovial tissue in rheumatoid arthritis Arthritis Rheum 2008 16 1001 1009 10.1002/art.23386 18383392

[B25] Dominguez-Gutierrez PR Ceribelli A Satoh M Sobel ES Reeves WH Chan EKL Elevated STAT1 correlates with increased CCL2 and CXCL10 levels in peripheral blood of patients with systemic lupus erythematosus Arthritis Res Ther 2014 16 R20 10.1186/ar4448 24451065 PMC3978614

[B26] Guiducci C Gong M Xu Z Gill M Chaussabel D Meeker T Chan JH Wright T Punaro M Bolland S Soumelis V Banchereau J Coffman RL Pascual V Barrat FJ TLR recognition of self nucleic acids hampers glucocorticoid activity in lupus Nature 2010 16 937 941 10.1038/nature09102 20559388 PMC2964153

[B27] Bhattacharyya S Zhao Y Kay TW Muglia LJ Glucocorticoids target suppressor of cytokine signaling 1 (SOCS1) and type 1 interferons to regulate Toll-like receptor-induced STAT1 activation Proc Natl Acad Sci USA 2011 16 9554 9559 10.1073/pnas.1017296108 21606371 PMC3111275

[B28] Aittomaki S Pesu M Groner B Janne OA Palvimo JJ Silvennoinen O Cooperation among Stat1, glucocorticoid receptor, and PU.1 in transcriptional activation of the high-affinity Fc gamma receptor I in monocytes J Immunol 2000 16 5689 5697 10820245 10.4049/jimmunol.164.11.5689

[B29] Dall’era M Chakravarty EF Treatment of mild, moderate, and severe lupus erythematosus: focus on new therapies Curr Rheumatol Rep 2011 16 308 316 10.1007/s11926-011-0186-6 21584692 PMC4400849

[B30] Chaturvedi A Pierce SK How location governs toll-like receptor signaling Traffic 2009 16 621 628 10.1111/j.1600-0854.2009.00899.x 19302269 PMC2741634

[B31] Takeda K Kaisho T Akira S Toll-like receptors Annu Rev Immunol 2003 16 335 376 10.1146/annurev.immunol.21.120601.141126 12524386

[B32] Tan EM Cohen AS Fries JF Masi AT McShane DJ Rothfield NF Schaller JG Talal N Winchester RJ The 1982 revised criteria for the classification of systemic lupus erythematosus Arthritis Rheum 1982 16 1271 1277 10.1002/art.1780251101 7138600

[B33] Nahid MA Yao B Dominguez-Gutierrez PR Kesavalu L Satoh M Chan EKL Regulation of TLR2-mediated tolerance and cross-tolerance through IRAK4 modulation by miR-132 and miR-212 J Immunol 2013 16 1250 1263 10.4049/jimmunol.1103060 23264652 PMC3552145

[B34] Livak KJ Schmittgen TD Analysis of relative gene expression data using real-time quantitative PCR and the 2(−Delta Delta C(T)) Method Methods 2001 16 402 408 10.1006/meth.2001.1262 11846609

[B35] Herbst R Liu Z Jallal B Yao Y Biomarkers for systemic lupus erythematosus Int J Rheum Dis 2012 16 433 444 10.1111/j.1756-185X.2012.01764.x 23083033

[B36] Hanley JA Negassa A Edwardes MD Forrester JE Statistical analysis of correlated data using generalized estimating equations: an orientation Am J Epidemiol 2003 16 364 375 10.1093/aje/kwf215 12578807

[B37] Crow MK Kirou KA Wohlgemuth J Microarray analysis of interferon-regulated genes in SLE Autoimmunity 2003 16 481 490 10.1080/08916930310001625952 14984025

[B38] Kirou KA Lee C George S Louca K Papagiannis IG Peterson MG Ly N Woodward RN Fry KE Lau AY Prentice JG Wohlgemuth JG Crow MK Coordinate overexpression of interferon-alpha-induced genes in systemic lupus erythematosus Arthritis Rheum 2004 16 3958 3967 10.1002/art.20798 15593221

[B39] Nikpour M Dempsey AA Urowitz MB Gladman DD Barnes DA Association of a gene expression profile from whole blood with disease activity in systemic lupus erythaematosus Ann Rheum Dis 2008 16 1069 1075 10.1136/ard.2007.074765 18063674

[B40] Qing X Putterman C Gene expression profiling in the study of the pathogenesis of systemic lupus erythematosus Autoimmun Rev 2004 16 505 509 10.1016/j.autrev.2004.07.001 15546798

[B41] Rhen T Cidlowski JA Antiinflammatory action of glucocorticoids-new mechanisms for old drugs N Engl J Med 2005 16 1711 1723 10.1056/NEJMra050541 16236742

[B42] Hu X Li WP Meng C Ivashkiv LB Inhibition of IFN-gamma signaling by glucocorticoids J Immunol 2003 16 4833 4839 12707366 10.4049/jimmunol.170.9.4833

[B43] Fulkerson PC Zimmermann N Hassman LM Finkelman FD Rothenberg ME Pulmonary chemokine expression is coordinately regulated by STAT1, STAT6, and IFN-gamma J Immunol 2004 16 7565 7574 15585884 10.4049/jimmunol.173.12.7565

[B44] Kok SH Hong CY Kuo MY Wang CC Hou KL Lin YT Galson DL Lin SK Oncostatin M-induced CCL2 transcription in osteoblastic cells is mediated by multiple levels of STAT-1 and STAT-3 signaling: an implication for the pathogenesis of arthritis Arthritis Rheum 2009 16 1451 1462 10.1002/art.24452 19404962

[B45] Valente AJ Xie JF Abramova MA Wenzel UO Abboud HE Graves DT A complex element regulates IFN-gamma-stimulated monocyte chemoattractant protein-1 gene transcription J Immunol 1998 16 3719 3728 9759897

[B46] Ansari AW Schmidt RE Heiken H Prednisolone mediated suppression of HIV-1 viral load strongly correlates with C-C chemokine CCL2: in vivo and in vitro findings Clin Immunol 2007 16 1 4 10.1016/j.clim.2007.07.003 17707134

[B47] Matsuo H Tamura M Kabashima N Serino R Tokunaga M Shibata T Matsumoto M Aijima M Oikawa S Anai H Nakashima Y Prednisolone inhibits hyperosmolarity-induced expression of MCP-1 via NF-kappaB in peritoneal mesothelial cells Kidney Int 2006 16 736 746 10.1038/sj.ki.5000131 16518329

[B48] de Kruif MD Lemaire LC Giebelen IA Groot AP Pater JM van den Pangaart PS Elliott PJ van der Poll T Effects of prednisolone on the systemic release of mediators of cell-mediated cytotoxicity during human endotoxemia Shock 2008 16 458 461 17909456 10.1097/shk.0b013e3181598a6a

[B49] Khodarev NN Roizman B Weichselbaum RR Molecular pathways: interferon/stat1 pathway: role in the tumor resistance to genotoxic stress and aggressive growth Clin Cancer Res 2012 16 3015 3021 10.1158/1078-0432.CCR-11-3225 22615451

[B50] Khodarev NN Beckett M Labay E Darga T Roizman B Weichselbaum RR STAT1 is overexpressed in tumors selected for radioresistance and confers protection from radiation in transduced sensitive cells Proc Natl Acad Sci USA 2004 16 1714 1719 10.1073/pnas.0308102100 14755057 PMC341831

[B51] Khodarev NN Minn AJ Efimova EV Darga TE Labay E Beckett M Mauceri HJ Roizman B Weichselbaum RR Signal transducer and activator of transcription 1 regulates both cytotoxic and prosurvival functions in tumor cells Cancer Res 2007 16 9214 9220 10.1158/0008-5472.CAN-07-1019 17909027

[B52] Kita K Sugaya S Zhai L Wu YP Wano C Chigira S Nomura J Takahashi S Ichinose M Suzuki N Involvement of LEU13 in interferon-induced refractoriness of human RSa cells to cell killing by X rays Radiat Res 2003 16 302 308 10.1667/RR3039 12926988

[B53] Rickardson L Fryknas M Dhar S Lovborg H Gullbo J Rydaker M Nygren P Gustafsson MG Larsson R Isaksson A Identification of molecular mechanisms for cellular drug resistance by combining drug activity and gene expression profiles Br J Cancer 2005 16 483 492 10.1038/sj.bjc.6602699 16012520 PMC2361589

[B54] Roberts D Schick J Conway S Biade S Laub PB Stevenson JP Hamilton TC O’Dwyer PJ Johnson SW Identification of genes associated with platinum drug sensitivity and resistance in human ovarian cancer cells Br J Cancer 2005 16 1149 1158 10.1038/sj.bjc.6602447 15726096 PMC2361951

[B55] de Prati AC Ciampa AR Cavalieri E Zaffini R Darra E Menegazzi M Suzuki H Mariotto S STAT1 as a new molecular target of anti-inflammatory treatment Curr Med Chem 2005 16 1819 1828 10.2174/0929867054546645 16101503

