# Supplementary material for: Reduced levels of CCL2 and CXCL10 in systemic lupus erythematosus patients under treatment with prednisone, mycophenolate mofetil, or hydroxychloroquine, except in a high STAT1 subset
Source: Arthritis Res Ther. 2014 Jan 24;16(1):R23. doi: 10.1186/ar4451 (PMC3978465; doi:10.1186/ar4451)
Supplement: Additional file 1: Figure S1 — Expression of different biomarkers in high versus low STAT1 populations in both SLE and healthy donors. Figure S2. Comparison of high and low STAT1 subsets of all treated to untreated SLE patient visits. Figure S3. Comparison of high and low STAT1 subsets of PDN treated patient visits to untreated patient visits. Figure S4. Comparison of high and low STAT1 subsets of HCQ treated patient visits to untreated patient visits. Figure S5. Comparison of high and low STAT1 subsets of MMF treated patient visits to untreated patient visits. Figure S6. Comparison of high and low STAT1 and dosage subsets on expression levels of the various biomarkers in the SLE cohort. Figure S7. Separate analyses of high and low STAT1 effects on CCL2 expression in various combined therapies. Figure S8. Separate analyses of high and low STAT1 effects on CXCL10 expression in various combined therapies. [file ar4451-S1.pdf]

Reduced levels of CCL2 and CXCL10 in SLE patients under treatment with prednisone, mycophenolate mofetil, or hydroxychloroquine, except in a high STAT1 subset

Paul R. Dominguez-Gutierrez[1], Angela Ceribelli[1], Minoru Satoh[2,3], Eric S. Sobel[2], Westley H. Reeves[2] and Edward K.L. Chan[1*]

[1]Department of Oral Biology, University of Florida, P.O. Box 100424, 1395 Center Drive, Gainesville, FL 32610-0424, USA, [22]Division of Rheumatology and Clinical Immunology, Department of Medicine, [3]Department of Pathology, Immunology, and Laboratory Medicine, University of Florida, P.O. Box 100221, 1600 SW Archer Rd, Gainesville, FL 32610-0221, USA

Additional file 1
Figure S1-8

Figure S1

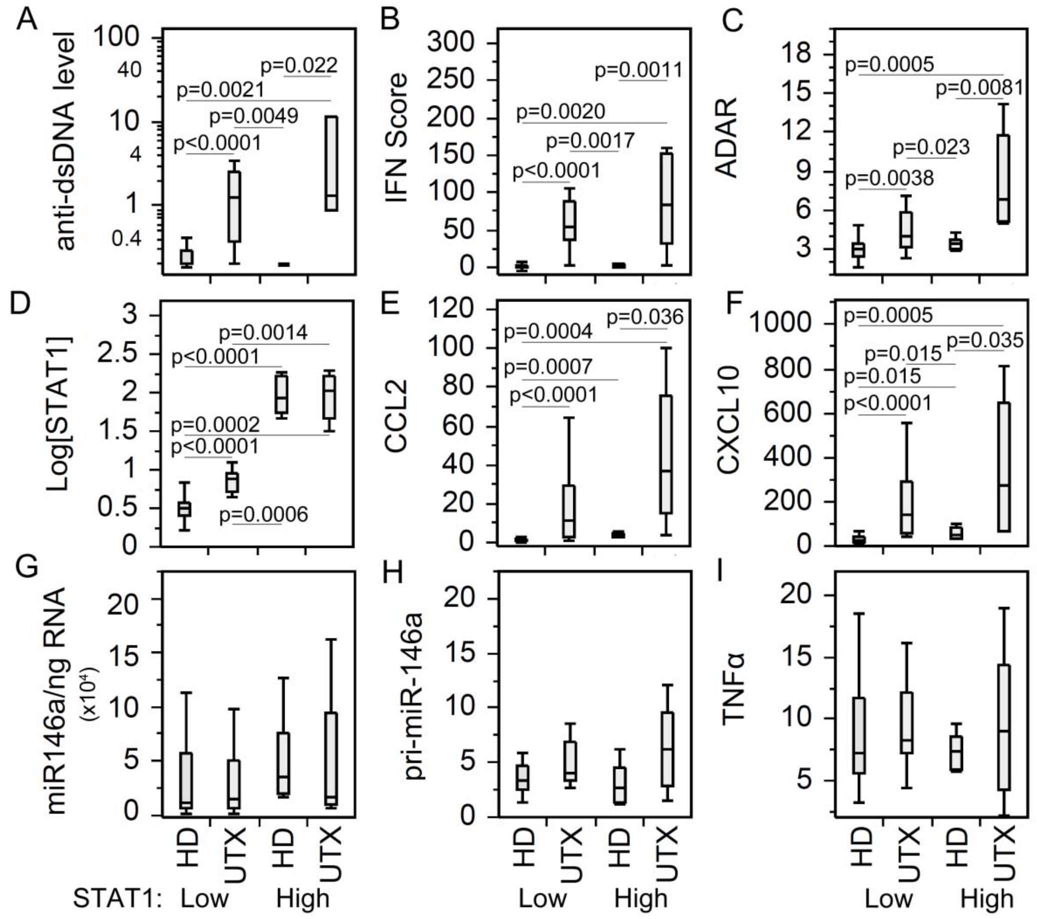

Expression of different biomarkers in high versus low STAT1 populations in both SLE and healthy donors. When analyzing different candidate biomarkers, there was no significant difference between high and low STAT1 UTX patients, with the exception of STAT1 (**D**). Comparing high and low STAT1 HD showed significant difference in STAT1 (**D**), CCL2 (**E**), and CXCL10 (**F**) but no significant difference for anti-dsDNA (**A**), IFN score (**B**), ADAR (**C**), miR-146a (**G**), pri-miR-146a (**H**), and TNFα (**I**). Regardless of STAT1 levels, UTX patients were significantly higher in anti-dsDNA, IFN score, ADAR, STAT1, CCL2, and CXCL10 than HD (**A-F**). Data are presented in box plot. All groups were compared among each other and only significant *P* values are shown indicating each specific comparison.

Figure S2

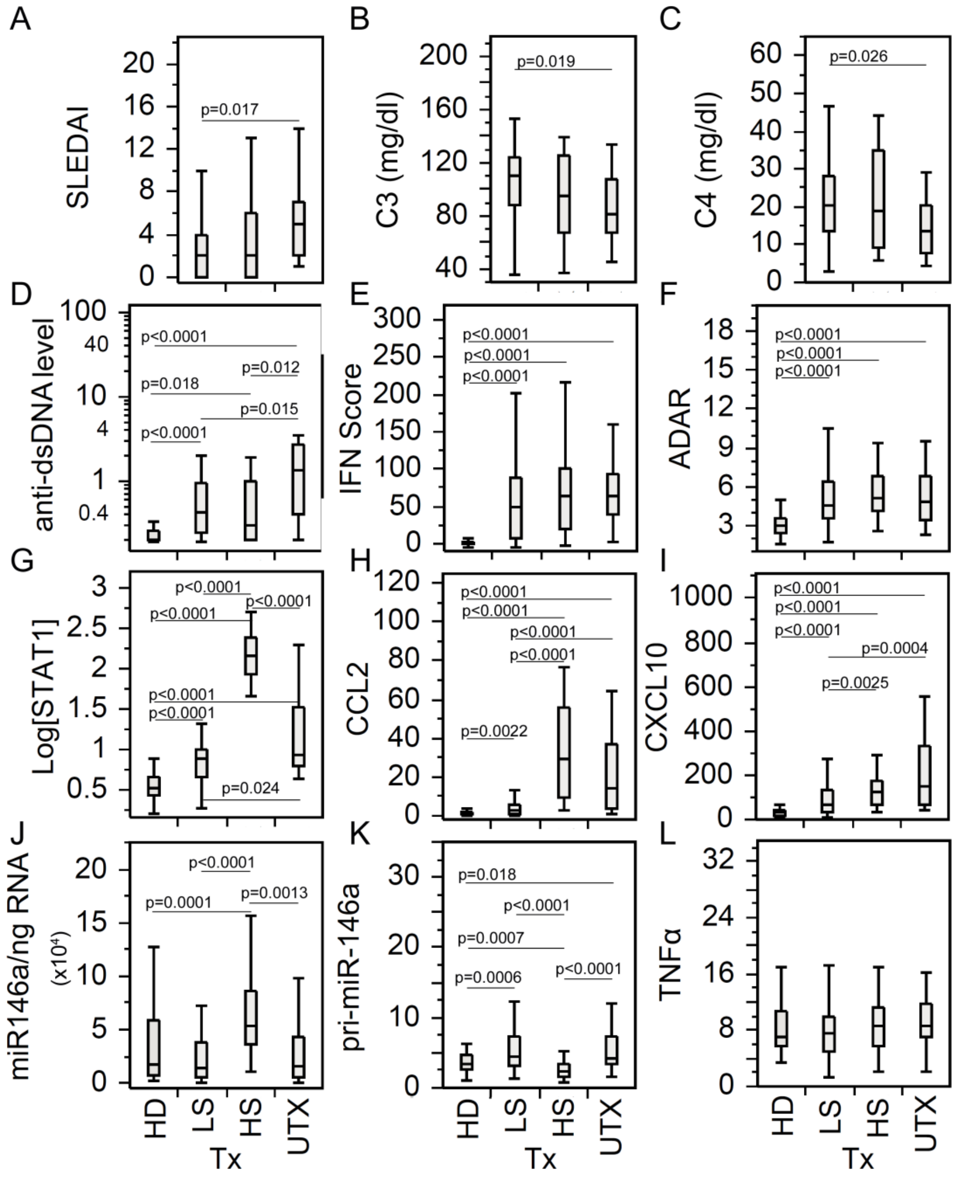

Comparison of high and low STAT1 subsets of all treated to untreated SLE patient visits. Data from Figure 1 are replotted to examine significant differences between the high and low STAT1 subsets.

Figure S3

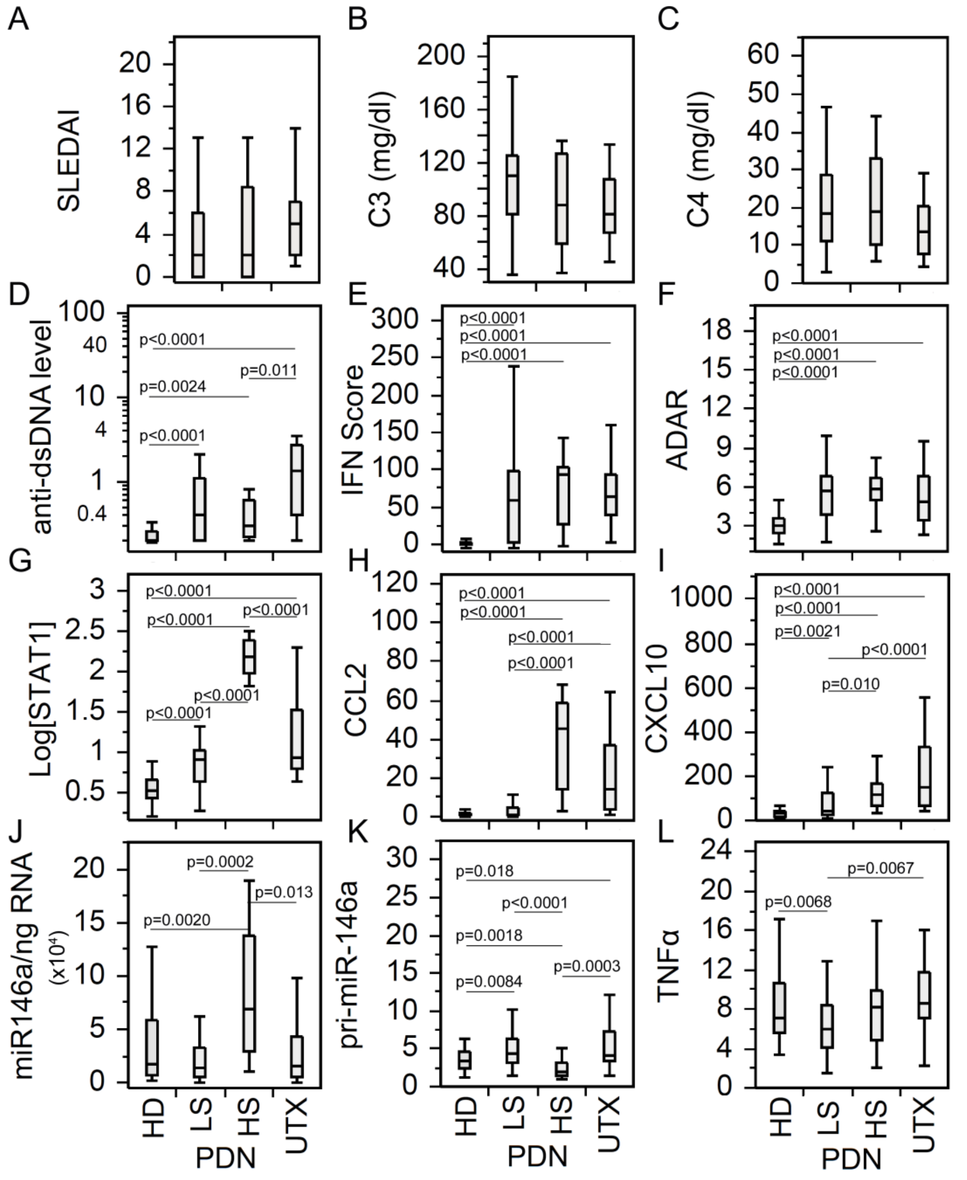

Comparison of high and low STAT1 subsets of PDN treated patient visits to untreated patient visits. Data from Figure 2 are replotted to examine significant differences between the high and low STAT1 subsets.

Figure S4

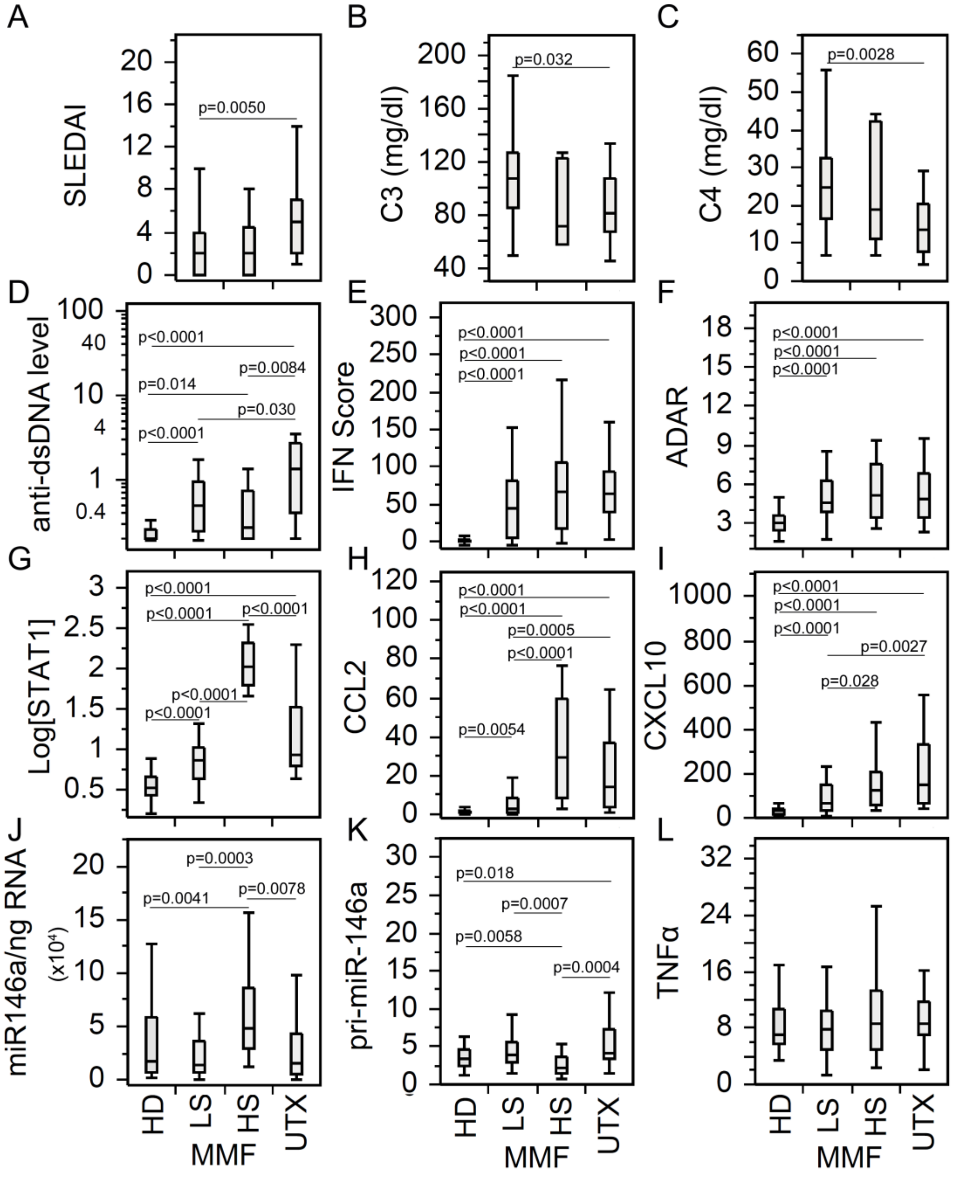

Comparison of high and low STAT1 subsets of HCQ treated patient visits to untreated patient visits. Data from Figure 3 are replotted to examine significant differences between the high and low STAT1 subsets.

Figure S5

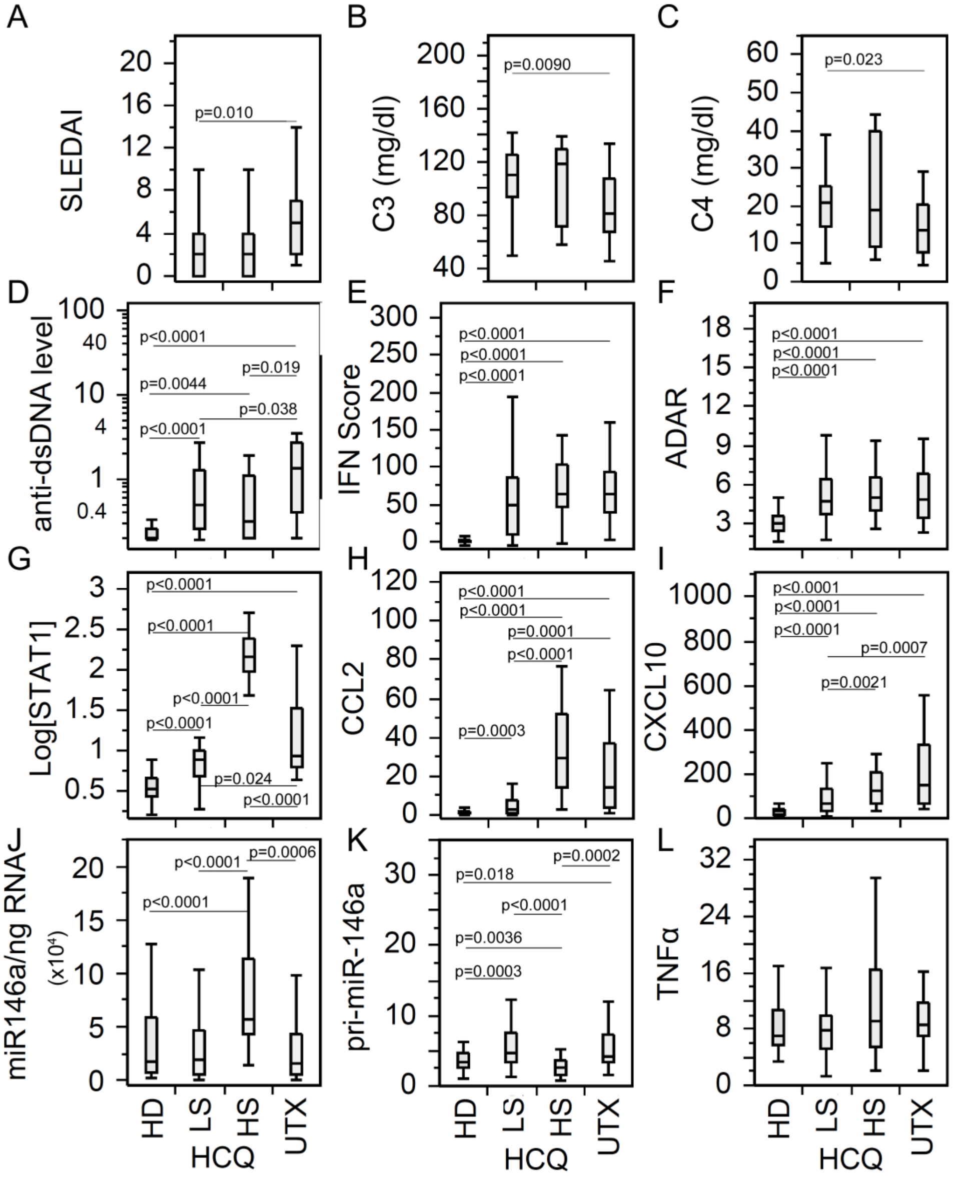

Comparison of high and low STAT1 subsets of MMF treated patient visits to untreated patient visits. Data from Figure 4 are replotted to examine significant differences between the high and low STAT1 subsets.

Figure S6

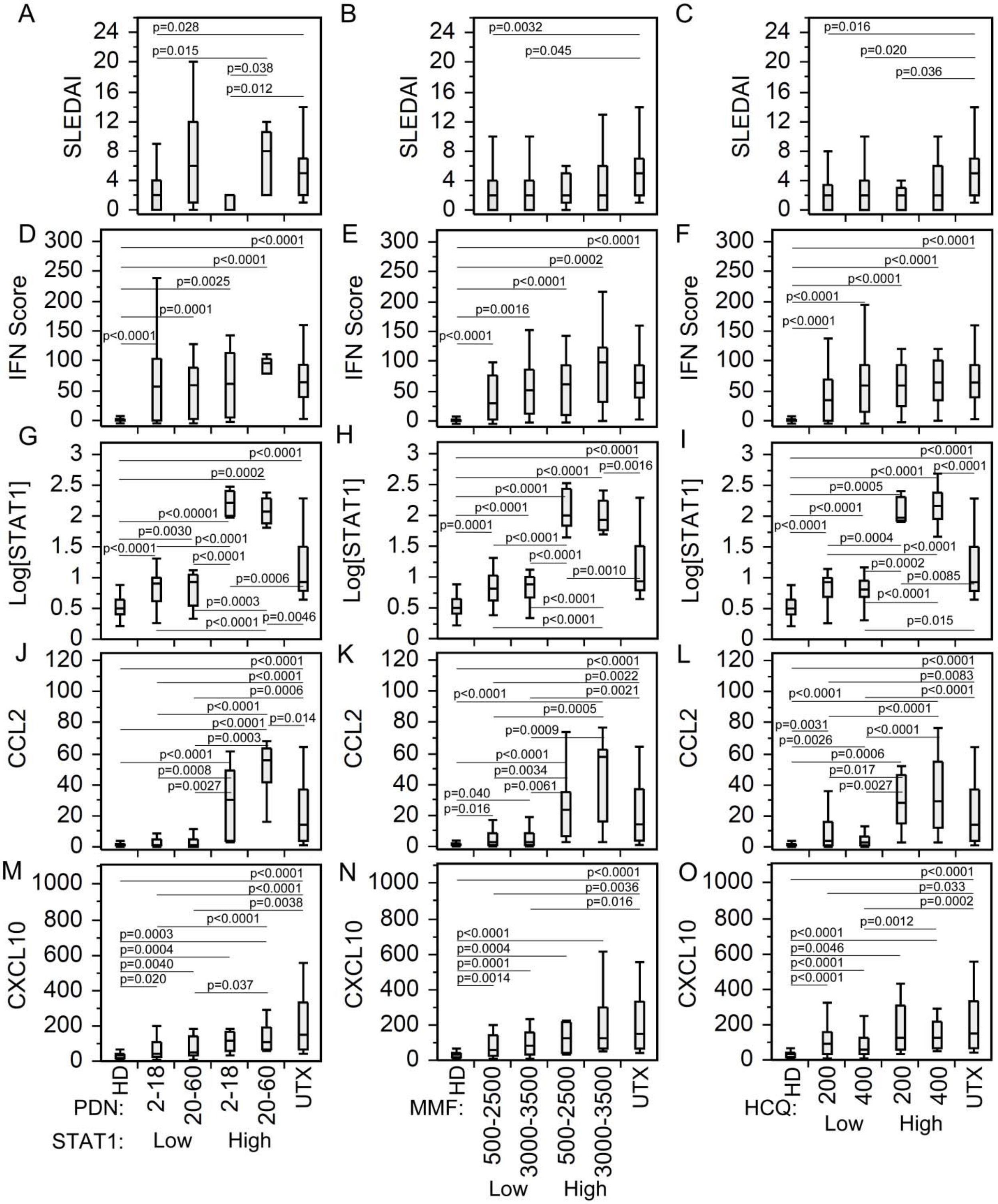

Comparison of high and low STAT1 and dosage subsets on expression levels of the various biomarkers in the SLE cohort. Data from Figure 5 are replotted to examine significant differences between the high and low STAT1 subsets.

Figure S7

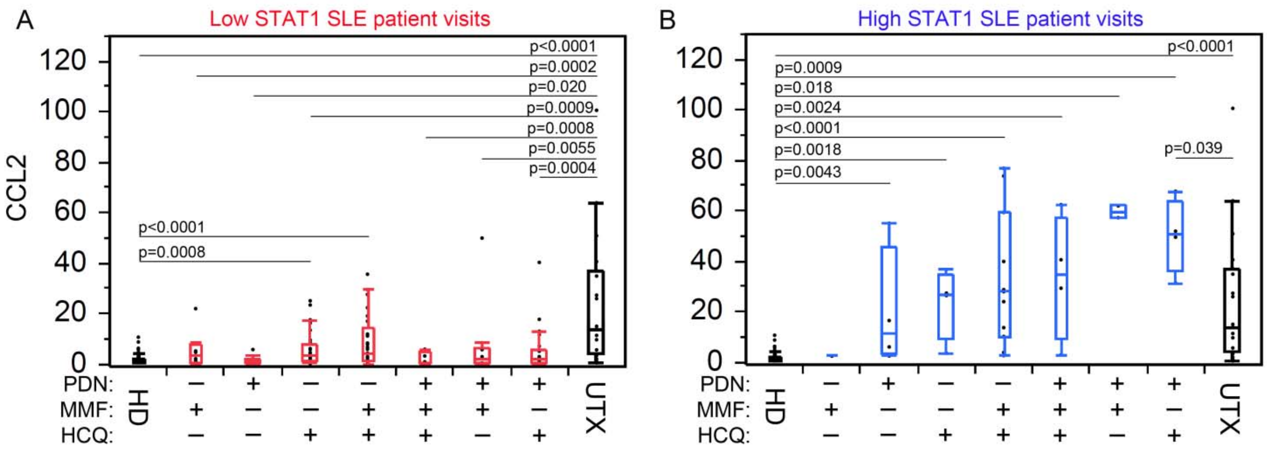

Separate analyses of high and low STAT1 effects on CCL2 expression in various combined therapies. Data from Figure 8A are replotted to examine significant differences between the high and low STAT1 subsets.

Figure S8

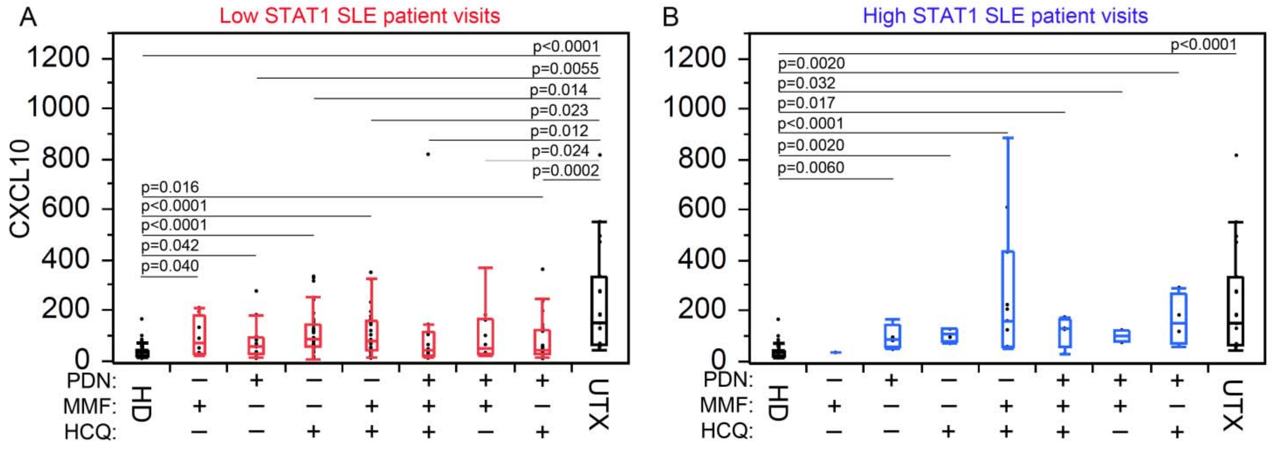

Separate analyses of high and low STAT1 effects on CXCL10 expression in various combined therapies. Data from Figure 8B are replotted to examine significant differences between the high and low STAT1 subsets.
